# Prevalence of Early Childhood Caries Among Children Visiting the Outpatient Department of a Dental Hospital in Meerut City: An Institutional-Based Cross-Sectional Study

**DOI:** 10.7759/cureus.75067

**Published:** 2024-12-03

**Authors:** Navpreet Kaur, Nikhil Srivastava, Vivek Rana, Noopur Kaushik, Tushar Pruthi, Aarushi kankerwal, Riya Chaudhary

**Affiliations:** 1 Pediatric and Preventive Dentistry, Subharti Dental College and Hospital, Swami Vivekanand Subharti University, Meerut, IND

**Keywords:** children, decayed missing and filled teeth (dmft), dental caries, early childhood caries, prevalence

## Abstract

Background: Early childhood caries (ECC) represents a significant public health challenge, characterized by the rapid decay of primary teeth in young children. This condition adversely affects oral health, overall well-being, and quality of life (QoL).

Aim: The study aimed to assess the prevalence of ECC among children attending a dental outpatient department in Meerut City.

Materials and methods: An institute-based cross-sectional study was conducted among children aged 2-6 years who visited the dental outpatient department (OPD) of the Pediatric and Preventive Dentistry Department. A total of 1800 children were examined using Type III examination (employing a mouth mirror and explorer under proper illumination). Data were recorded using the WHO Oral Health Assessment Pro Forma (1997). The Decayed, Missing, and Filled Teeth (DMFT) index was used to evaluate dental caries. Data were tabulated based on gender, age, DMFT score, and symptoms. Statistical analysis was conducted using IBM SPSS Statistics software version 23, with the level of significance set at P < 0.05. Descriptive data were presented as percentages, and Fisher’s exact test was employed for statistical analysis.

Results: Of the 1800 children examined, 1086 were found to have ECC, yielding a prevalence of 60.3%. Among the affected children, 61.6% were males, and 38.4% were females. ECC prevalence was significantly higher in children aged 3-6 years compared to those under 3 years (P = 0.001). The mean DMFT score of the study population was 4.27 ± 2.62, with the highest prevalence observed for a DMFT score of 4. The frequency of ECC was higher in patients who presented to the OPD with pain (70.9%).

Conclusion: The prevalence of ECC among children aged 2-6 years who visited the dental OPD was 60.3%, with a mean DMFT score of 4.27 ± 2.62. The prevalence was higher in male children compared to female children. Additionally, the most common reason for visiting the dental OPD was pain.

## Introduction

Early childhood caries (ECC) is a major dental health problem affecting young children globally, presenting significant challenges to dental professionals and caregivers. It is defined as the presence of decayed, missing, or filled dental surfaces in children below the age of six [1]. Factors such as prolonged exposure to sugary beverages, frequent consumption of sweet snacks, poor oral hygiene habits, bacterial infections (e.g., *Streptococcus mutans*), and limited access to dental care contribute to its development [2].

ECC often leads to rapid decay in primary teeth, causing symptoms such as pain, infection, difficulty in brushing, challenges in chewing, prolonged retention of food in the mouth, and long-term dental complications like tooth abscesses and recurrent fevers. It predominantly affects infants, toddlers, and preschool-aged children. Despite being a preventable condition, ECC remains highly overlooked in India, negatively impacting the overall health and quality of life of affected children [3]. If left untreated, ECC can result in developmental issues with permanent teeth, poor aesthetics, speech difficulties, and malocclusions. Additionally, children with ECC often miss school due to low self-esteem associated with decayed or missing teeth [3]. Recognizing the causes, effects, and preventive measures for ECC is therefore critical for both caregivers and dental professionals.

Globally, ECC has reached epidemic proportions, though its prevalence varies across populations. The highest rates are reported in Far East Asia, where 36-85% of three-year-olds are affected [4]. In India, studies indicate that 44% of children aged 8-48 months are affected by ECC [5]. In rural South India, around 40.6% of children under three years have ECC, with 50.3% presenting with non-cavitated lesions and 49.7% with cavitated surfaces [6]. Maxillary central incisors and molars are the most commonly affected teeth, while mandibular central incisors are typically spared due to the protective salivary action of submandibular and sublingual glands and the tongue's coverage of these teeth [7].

Addressing ECC is vital not only for oral health but also for the overall well-being of affected children. Early intervention and preventive strategies are crucial to ensuring better health outcomes and maintaining children's smiles. Prioritizing disease control is a societal necessity, particularly for identifying high-risk populations and implementing targeted interventions.

Understanding the prevalence of ECC in young children is essential in Meerut, a major district in Western Uttar Pradesh with a population exceeding 3.39 million, including approximately 500,000 children under six. By identifying the extent of ECC in this population and the factors associated with its occurrence, healthcare providers can allocate resources effectively and develop focused interventions to improve oral health outcomes for children in the region. The present research aimed to evaluate the prevalence and experience of ECC among children visiting the Dental Outpatient Department (OPD) at Subharti Dental College and Hospital in Meerut.

## Materials and methods

The research was a cross-sectional investigation conducted on children who attended the Pediatric and Preventive Dentistry Department at Subharti Dental College and Hospital. Ethical clearance was obtained from University Ethics Committee (Medical), Swami Vivekanand Subharti University prior to the study (Ref No: SMC/UECM/2024/957). The study was carried out between April 2024 and September 2024 and included all children aged 2-6 years visiting the OPD for dental treatment.

Study population and sampling design

The study targeted children who visited the dental outpatient department (OPD) of the Pediatric and Preventive Dentistry Department. A convenience sampling method was used to select participants. Children aged 2-6 years attending the OPD were included in the study, provided parental and institutional consent was obtained. Children with systemic disorders, oral diseases, or conditions that impeded oral examination, as well as those who were uncooperative during the examination, were excluded from participation.

The sample size was calculated using the following formula:

N = Z^2^pq/d^2^

where N is the sample size, Z = 1.96 is the point of normal distribution (as per the table of the area under the normal curve for the given confidence level of 95%), p = 19% is the proportion or prevalence of interest [8], q = 100 - p (alternative prevalence), and d = 2% is the clinically acceptable error.

Applying the formula for this study,

N = 1.96 × 1.96 (19 (100 - 19))/2 × 2 = 1,478

Clinical examination

The examinations were conducted at the Outpatient Department of Pediatric and Preventive Dentistry at Subharti Dental College and Hospital, Swami Vivekanand Subharti University, Meerut. Each child underwent a Type III dental examination (involving the use of a mouth mirror and blunt probe under proper lighting). Data on dental health status were collected using the WHO Oral Health Assessment Pro Forma (1997). The OPD's dental examiners evaluated the DMFT (decayed, missing, and filled teeth) index. Interexaminer reliability for DMFT scoring was high, with a kappa coefficient of 0.88. Caries experience was assessed by calculating the average number of teeth that were decayed, missing, or filled.

Statistical analysis

The collected data were organized and analyzed using IBM SPSS Statistics for Windows, Version 23 (Released 2015; IBM Corp., Armonk, New York). The Fisher Exact test was applied to compare caries experience between males and females. Percentages were used to present the descriptive statistics, with P < 0.05 considered statistically significant.

## Results

Out of a total of 1800 children examined, ECC was present in 1086 subjects, resulting in a prevalence of 60.3%. Among the 1086 children with ECC, 669 were males and 417 were females. The prevalence of ECC among males and females was 61.6% and 38.4%, respectively. The difference in ECC prevalence between male and female subjects was found to be statistically significant. As age increased, so did the prevalence of ECC, with the highest occurrence (93%) observed among children aged 3 to 6 years, compared to 6.8% in children younger than three years. A significantly higher prevalence of ECC was noted in the 3-6-year-old age group compared to the under-3-year-old group (P < 0.05) (Table 1).

**Table 1:** Prevalence and comparison of early childhood caries (ECC) among male and female subjects and different age groups Fisher exact test; the asterisk (*) indicates significant difference at p<0.05.

Total population studied	Subjects with ECC	P-value
1800	1086 (60.3%)	
Gender		
Male	669 (61.6%)	0.019*
Female	417 (38.4%)	
Age group		
<3 years	74 (6.8%)	<0.001*
3-6 years	1012 (93%)	

For the study population, the mean DMFT score was 4.27 (Table 2). Older children had a significantly higher DMFT score, with a statistically significant mean score of 4.

**Table 2:** Mean DMFT (decayed, missing, and filled teeth) index among subjects with early childhood caries (ECC)

Variable	Minimum–maximum	Mean ± SD
ECC	1–17	4.27 ± 2.62

The primary reason for visiting the OPD was pain (70.9%), followed by tooth decay (14.4%), swelling (9.4%), and other complaints (5.3%) (Table 3).

**Table 3:** Details of reasons for seeking treatment

Reason	N (%)
Decayed	156 (14.4%)
Pain	770 (70.9%)
Sinus	102 (9.4%)
Others	58 (5.3%)

## Discussion

The objective of this research was to evaluate the prevalence of ECC among children aged 2 to 6 years in Meerut, a district in Uttar Pradesh, and to analyze associated factors such as gender differences, severity of ECC (measured by the mean DMFT score), and reasons for seeking dental care. Among the 1800 children who met the inclusion criteria, the prevalence of ECC in children attending the dental outpatient department was found to be 60.3%. Comparatively, other studies conducted in India on children from various populations reported ECC prevalence rates of 27.5% (Bengaluru urban, Karnataka), 32% (Rohtak, Haryana), 42.03% (Bahadurgarh, Haryana), 54.1% (Hubli-Dharwad City, Karnataka), and 47.2% in Bhubaneswar [9].

The quality of this study was evaluated using the Newcastle-Ottawa Scale, a widely accepted tool for assessing the quality of observational studies, particularly in non-randomized designs, which justifies the rigor and reliability of the study's methodology.

Consistent with the guidelines of the American Academy of Pediatric Dentistry (AAPD), only children up to 6 years of age were included in the investigation [1]. The AAPD guidelines emphasize the importance of focusing on children in early childhood for dental health assessments, particularly for conditions like ECC [1].

Various methods have been employed to measure dental caries across studies, with the most common being the DMFT index [10]. For over 70 years, the Decayed, Missing, and Filled Teeth (DMFT) index has been globally recognized as a crucial tool for assessing oral and dental health. This index quantifies the number of decayed, treated, and missing teeth due to decay [10]. It also plays an essential role in evaluating and monitoring oral health interventions, aiding in the development of community policies and programs [10].

Most existing research on ECC prevalence has been concentrated in Karnataka, with fewer studies from other regions of India. This geographic imbalance limits the generalizability of ECC prevalence data to the broader population of the country [5].

Globally, ECC prevalence among children aged 3-5 years varies widely depending on the region and country. Several factors contribute to the elevated rates of dental caries in this age group, including inadequate oral hygiene practices, limited awareness of oral health importance, and neglect by caregivers. Socioeconomic status (SES) also plays a significant role, with lower SES being associated with higher levels of cariogenic bacteria and increased stress, as indicated by elevated salivary cortisol levels [11]. The lack of affordable and accessible dental care further exacerbates the high prevalence of ECC.

In the current research, the mean DMFT score was 4.27, with ECC being more prevalent among males (61.6%) than females (38.4%). The higher prevalence of ECC among male children may be attributed to socio-cultural practices in the rural areas surrounding the study location, where male children are often prioritized over females. Consequently, boys are more frequently provided with cariogenic foods and beverages. Additionally, male children may exhibit less consistent oral hygiene practices due to reduced emphasis on oral health compared to their female counterparts. However, previous studies have reported mixed results regarding the influence of gender on caries prevalence, indicating the need for further research to clarify these discrepancies [12-14].

The study also identified a marked increase in ECC prevalence as children aged, consistent with earlier research findings. This age-related increase may result from prolonged exposure of teeth to the oral environment and evolving dietary habits as children grow [15,16].

This study revealed that most children were brought to dental clinics due to pain, followed by decayed teeth. Similar findings were reported by Olatosi et al., where 33.1% of children visited dental clinics due to pain. In contrast, research by Daou et al. found that the primary reasons for dental visits were the presence of decayed teeth (50.9%) and perceived dental pain (29.5%) [17,18]. Studies by Yahya et al., Soxman, and Masiga have also concluded that dental caries and associated complications were the predominant causes of children’s dental visits [19-21].

Pain being the most common reason for dental visits among children is primarily due to parents failing to recognize early signs of dental issues, such as caries, gingivitis, or other oral conditions, until they progress to pain. These issues can be effectively addressed through prevention and early intervention strategies, including good oral hygiene practices, limiting sugary snack consumption, regular dental visits, and fluoride treatments. Public awareness about ECC prevention can also be enhanced through education campaigns, community outreach programs, school initiatives, and mass media efforts promoting oral hygiene and routine dental check-ups.

Limitations of the study

This study did not account for several risk factors that could influence ECC, such as breastfeeding history, dietary habits, oral hygiene practices, and social determinants (e.g., family income, number of siblings, parental occupation). These factors could have provided a more comprehensive understanding of ECC prevalence and risk. Future studies incorporating these variables are recommended to build upon the findings of this research.

## Conclusions

This study found that the prevalence of ECC among children aged 2-6 years who visited the dental OPD was 60.3%, with a mean DMFT score of 4.27 ± 2.62. The prevalence of ECC was notably higher in male children compared to female children, and it was most commonly observed in children aged 3-6 years compared to those under 3 years of age. Pain emerged as the primary reason for seeking dental care at the OPD. These findings emphasize the need for more comprehensive population-based studies, particularly in regions where data on ECC are limited. Expanding the scope of such research can provide a better understanding of ECC prevalence and inform the development of tailored public health strategies to address this significant dental health concern.

## References

[1] American Academy of Pediatric Dentistry (2023). Policy on early childhood caries (ECC): consequences and preventive strategies. The Reference Manual of Pediatric Dentistry.

[2] Xu H, Ma X, Wang J, Chen X, Zou Q, Ban J (2024). Exploring the state and influential factors of dental caries in preschool children aged 3-6 years in Xingtai City. BMC Oral Health.

[3] Sharma I, Sujlana A, Kour S, Sharma S (2024). Impact of ECC on the oral health-related quality of life of preschool children and their families. Int J Adv Res.

[4] Devan I, Ramanarayanan V, Janakiram C (2022). Prevalence of early childhood caries in India: a systematic review and meta-analysis. Indian J Public Health.

[5] Ganesh A, Muthu MS, Mohan A, Kirubakaran R (2019). Prevalence of early childhood caries in India - a systematic review. Indian J Pediatr.

[6] Henry JA, Muthu MS, Saikia A, Asaithambi B, Swaminathan K (2017). Prevalence and pattern of early childhood caries in a rural South Indian population evaluated by ICDAS with suggestions for enhancement of ICDAS software tool. Int J Paediatr Dent.

[7] Alazmah A (2017). Early childhood caries: a review. J Contemp Dent Pract.

[8] Gupta D, Momin RK, Mathur A (2015). Dental caries and their treatment needs in 3-5 year old preschool children in a rural district of India. N Am J Med Sci.

[9] Yavagal PC, Velangi CS, Singh I, Desai P, Sunny CH (2020). Prevalence of early childhood caries among children attending anganwadis in Davangere City: a cross sectional survey. J Indian Assoc Public Health Dent.

[10] Moradi G, Mohamadi Bolbanabad A, Moinafshar A, Adabi H, Sharafi M, Zareie B (2019). Evaluation of oral health status based on the decayed, missing and filled teeth (DMFT) index. Iran J Public Health.

[11] Boyce WT, Den Besten PK, Stamperdahl J, Zhan L, Jiang Y, Adler NE, Featherstone JD (2010). Social inequalities in childhood dental caries: the convergent roles of stress, bacteria and disadvantage. Soc Sci Med.

[12] Dixit A, Aruna DS, Sachdev V, Sharma A (2015). Prevalence of dental caries and treatment needs among 3-5 year old preschool children in Narmada, Gujarat. IOSR J Dent Med Sci.

[13] Virdi M, Bajaj N, Kumar A (2009). Prevalence of severe early childhood caries in pre‑school children in Bahadurgarh, Haryana, India. Internet J Epidemiol.

[14] Kashetty MV, Patil S, Kumbhar S, Patil P (2016). Prevalence of dental caries among 3-6 year old Anganwadi children in Mudhol town, Karnataka, India. J Indian Assoc Public Health Dent.

[15] Prakash P, Subramaniam P, Durgesh BH, Konde S (2012). Prevalence of early childhood caries and associated risk factors in preschool children of urban Bangalore, India: A cross sectional study. Eur J Dent.

[16] Stephen A, Krishnan R, Ramesh M, Kumar VS (2015). Prevalence of early childhood caries and its risk factors in 18-72 month old children in Salem, Tamil Nadu. J Int Soc Prev Community Dent.

[17] Olatosi OO, Inem V, Sofola OO, Prakash P, Sote EO (2015). The prevalence of early childhood caries and its associated risk factors among preschool children referred to a tertiary care institution. Niger J Clin Pract.

[18] Daou MH, Eden E, El Osta N (2016). Age and reasons of the first dental visit of children in Lebanon. J Med Liban.

[19] Yahya MD, Ayman FMAO, Enas FHO (2012). Mean age and chief complaint of the Jordanian children on their first dental visit. Pak Oral Dental J.

[20] Soxman JA (2002). The first dental visit. Gen Dent.

[21] Masiga MA (2004). Socio-demographic characteristics and clinical features among patients attending a private paediatric dental clinic in Nairobi, Kenya. East Afr Med J.

